# Optimizing intravenous amoxicillin for outpatient parenteral antimicrobial therapy

**DOI:** 10.1093/jacamr/dlag165

**Published:** 2026-08-06

**Authors:** Zenaw T Wolie, Jason A Roberts, Steven C Wallis, Getnet M Assefa, R Andrew Seaton, Mark Gilchrist, Fekade B Sime

**Affiliations:** Frazer Institute, Faculty of Health, Medicine and Behavioural Sciences, The University of Queensland, Brisbane, Queensland, Australia; Department of Pharmacy, College of Medicine and Health Sciences, Debre Markos University, Debre Markos, Ethiopia; Frazer Institute, Faculty of Health, Medicine and Behavioural Sciences, The University of Queensland, Brisbane, Queensland, Australia; Herston Infectious Diseases Institute (HeIDI), Metro North Health, Brisbane, Queensland, Australia; Departments of Pharmacy and Intensive Care Medicine, Royal Brisbane and Women’s Hospital, Brisbane, Queensland, Australia; UR UM 103, University of Montpellier, Division of Anesthesia Critical Care and Emergency and Pain Medicine, Nimes University Hospital, Nimes, France; Frazer Institute, Faculty of Health, Medicine and Behavioural Sciences, The University of Queensland, Brisbane, Queensland, Australia; Frazer Institute, Faculty of Health, Medicine and Behavioural Sciences, The University of Queensland, Brisbane, Queensland, Australia; Department of Pharmacy, College of Medicine and Health Sciences, Wollo University, Dessie, Ethiopia; Department of Infectious Diseases, Queen Elizabeth University Hospital, Glasgow, UK; OPAT Initiative, British Society for Antimicrobial Chemotherapy (BSAC), Birmingham, UK; OPAT Initiative, British Society for Antimicrobial Chemotherapy (BSAC), Birmingham, UK; Department of Pharmacy/Infection, Imperial College Healthcare NHS Trust, London, UK; Department of Infectious Diseases, Imperial College London, London, UK; Frazer Institute, Faculty of Health, Medicine and Behavioural Sciences, The University of Queensland, Brisbane, Queensland, Australia; School of Pharmacy and Pharmaceutical Sciences, Faculty of Health, Medicine and Behavioural Sciences, The University of Queensland, Brisbane, Queensland, Australia

## Abstract

**Background and objectives:**

Poor aqueous stability limits the use of continuous infusion of intravenous (IV) amoxicillin in outpatient parenteral antimicrobial therapy. This study aimed to evaluate whether buffering improves amoxicillin stability and whether co-administration of probenecid enhances amoxicillin exposure.

**Methods:**

Amoxicillin solutions (4.17, 50, and 66.6 mg/mL) were prepared in 0.3% citrate-buffered saline (pH 7), water for injection, and 0.9% w/v saline, then stored at 2–8°C for 10 days, followed by 24 h at 32°C. Stability was assessed using a validated assay method and tested against the UK Yellow Cover Document (YCD) acceptance criteria. A population pharmacokinetic model was developed and used to perform Monte Carlo dosing simulations to evaluate the PTA (95% time above MIC) for various IV amoxicillin regimens, with and without probenecid.

**Results:**

Citrate buffering did not improve the stability of amoxicillin, and none of the tested conditions met YCD criteria. Co-administration of probenecid significantly increased the PTA for amoxicillin. Administration of 3–6 g of IV amoxicillin every 12 h, with 1 g of probenecid once daily, achieved ≥90% PTA against pathogens with MICs up to 4–8 mg/L depending on infusion durations. For MICs up to 16 mg/L, 4–6 g of IV amoxicillin every 12 h plus 2 g of probenecid once daily achieved a favourable PTA (>90%).

**Conclusions:**

Amoxicillin’s aqueous instability was not resolved by citrate buffering. However, for patients requiring IV therapy, probenecid-boosted intermittent and extended infusion regimens of amoxicillin appear to be promising alternatives that warrant further clinical investigation.

## Introduction

Amoxicillin is considered an attractive narrow-spectrum option for managing deep-seated streptococcal and enterococcal infections, such as infective endocarditis.^1,2^ It has a good oral bioavailability and is readily available as an oral formulation.^3^ At the standard oral doses (1 g 6-hourly), some studies report likely adequate exposure up to the clinical MIC breakpoint of 4 mg/L for Enterococci (0.5 mg/L for Streptococci),^4^ although target exposure in deep-seated infections is poorly defined.^5^ Even when considering traditional exposure targets, some subsets of patients may require higher oral doses, a typical example being those with obesity, for which the standard oral dose was shown to be inadequate to cover pathogens with MIC >1 mg/L.^6,7^ However, amoxicillin exhibits a saturable, dose-dependent absorption, which means that a relatively low proportion is absorbed at high doses. For example, based on data from Sjövall *et al*, the relative bioavailability at a higher dose of 3000 mg, compared with 375 mg, is approximately 53.5%^8^; i.e. at a high dose of 3000 mg, the dose normalized exposure in terms of AUC is only about 53.5% that of a lower dose of 375 mg. Furthermore, pharmacokinetic/pharmacodynamic (PK/PD) evidence showed that amoxicillin oral regimens (e.g. 1000 mg four times daily) achieve ≤85% PTA (50% *f*T > MIC) at a clinical breakpoint of 4 mg/L, with higher MICs requiring substantially higher oral doses.^4^ This has two key clinical implications: first, when higher doses are needed, the substantial proportion of the dose remaining in the gastrointestinal (GI) tract is likely to worsen the common GI side effects of amoxicillin; and second, there is a clear risk of sub-therapeutic exposure for pathogens with relatively high MICs in the susceptible range. Therefore, when a guaranteed systemic exposure is critical, such as in patients with persisting deep-seated infections who are at risk of underexposure, intravenous (IV) amoxicillin is preferred in clinical practice, although there are emerging data supporting an IV-to-oral switch in some patients.^2,9^ IV amoxicillin would also be preferred in patients with known or suspected malabsorption, for example, patients recovering from critical illness or those with diabetes or other conditions affecting gut motility and oral absorption.

A key limitation of IV amoxicillin is that patients often have to remain hospitalized for IV therapy. The usual intermittent dosing regimens are inconvenient for the outpatient parenteral antimicrobial therapy (OPAT) programme. Like other beta-lactam antimicrobials, continuous infusion (CI) of amoxicillin in OPAT is considered not feasible due to poor stability in aqueous solvents commonly used for powder reconstitution.^10–14^ The UK NHS ‘Yellow Cover Document’ (YCD) provides a standardized framework for assessing drug stability in infusion devices. It specifies testing an in-use temperature of up to 32 ± 1°C and recommends stability testing after reconstitution, including pre- or fresh-filling of devices, refrigerated storage (2–8°C), and a 24-h running phase at 32°C.^15^ However, most previous amoxicillin stability data were generated under non-YCD-compliant conditions, typically at room temperature (15–25°C) or higher, up to 37°C,^10,11,16,17^ limiting their practical relevance to OPAT conditions. Buffering strategies (such as 0.3% citrate at pH 7) have improved the stability of other β-lactams,^18^ yet remain uncharacterized for amoxicillin.

In parallel, given that optimizing amoxicillin stability has previously proven challenging,^19,20^ alternative approaches that would enable the use of IV amoxicillin in the OPAT programme warrant investigation. One such approach could be to leverage the PK interaction with probenecid to enable less frequent, short infusions in OPAT settings, thereby avoiding the stability concerns associated with CIs. Probenecid, by reducing renal tubular secretion of several β-lactams, has been shown to increase systemic exposure^21^ and may serve as a practical adjunct to improve the feasibility of amoxicillin use in OPAT without requiring CI.

Thus, the dual aims of this study are: (i) to evaluate the stability of amoxicillin buffered with 0.3% citrate at pH 7 under YCD-compliant OPAT conditions and (ii) to define effective probenecid-boosted IV amoxicillin dosing regimens for use in OPAT using a population PK (PopPK) model-guided *in silico* Monte Carlo dosing simulations.

## Materials and methods

### Stability study

#### Materials

Amoxicillin powder for injection (Fisamox, Batch: 2AA196006) was sourced from Aspen Pharmacare, New South Wales (NSW), Australia. A citrate-buffered saline (CBS) solution (0.3% w/v, pH 7) was prepared by dissolving trisodium citrate dihydrate and citric acid anhydrous (Sigma-Aldrich, Lots SLCG13355 and STBJ8530, Victoria, Australia) in 0.9% sodium chloride solution (Baxter Healthcare Pty. Ltd, NSW, Australia) and adjusting the pH to 7.0. HPLC-grade acetonitrile (Merck, Darmstadt, Germany) and LC-MS-grade methanol (Fisher Chemicals, Fair Lawn, USA) were used as the mobile phase. Analytical-reagent-grade disodium hydrogen phosphate (Merck, Darmstadt, Germany) was used to prepare the mobile-phase buffer. Ultrapure water was obtained from a Milli-Q Direct water purification system.

#### Sample preparation

Amoxicillin powder for injection was reconstituted in sterile borosilicate glass bottles using three diluents: 0.3% w/v CBS (pH 7), normal saline (0.9% NaCl), and unbuffered water for injection (WFI). Solutions were prepared at low (4.17 mg/mL), intermediate (50 mg/mL), and high (66.6 mg/mL) concentrations, corresponding to 1, 12, and 16 g in 240 mL in infusion devices, respectively. For each diluent, each concentration was prepared in triplicate and stored at 5 ± 3°C, protected from light, for 10 days. The samples were then incubated at the ‘in-use’ temperature (32°C) for 24 h without UV exposure. Serial samples (∼2 mL) were collected at 0, 12, 24, 48, 96, 168, and 240 h during refrigeration, and at 0, 2, 6, 8, 12, and 24 h during exposure to the ‘in-use’ temperature of 32°C. A 0.5 mL portion of each sample was immediately frozen at −80°C for concentration analysis. At the same time, the remaining volume was used for visual assessment of any precipitation and for pH measurement using an Orion semi-micro electrode and an Eutech pH700 meter (Eutech Instruments, Singapore).

#### Assay method and sample analysis

Amoxicillin concentrations were measured using a validated stability-indicating HPLC assay, confirmed by forced degradation (Figures S1–S3, available as Supplementary data at *JAC-AMR* Online). Analysis employed a Nexera X2 HPLC system coupled to SPD-M30A photodiode array detector (Shimadzu, Kyoto, Japan), using a Symmetry C18 (100 × 2.1 mm, 3.5 µm) analytical column preceded by a VanGuard Symmetry C18 (3.5 µm) guard cartridge (Waters, Milford, USA). The stationary phase was maintained at 30°C. Gradient elution (flow rate: 0.4 mL/min, producing a backpressure of 3000 psi) was applied using mobile phase A (20 mM phosphate buffer, pH 5.0) and mobile phase B (20% acetonitrile, 10% methanol, 40% water). The gradient was ramped from 4% B to 30% B over 9 min. At each concentration level, precision (%RSD) was ≤1.9%, accuracy was ≤0.4%, and correlation coefficients (*r*^2^) from three linearity batches were 0.99717, 0.99517, and 0.99917. Batch acceptance criteria were in accordance with the US FDA guidelines.

### PopPK modelling

#### Data sources and study population

Individual patient-level concentration–time data for amoxicillin, with and without co-administration of probenecid, were obtained from a previously published study involving healthy adult volunteers (aged 22–26 years; body weight 60–80 kg).^22^ All participants provided informed consent before enrolment. Each subject received either a single 3 g oral dose of amoxicillin or the same regimen co-administered with 1 g oral probenecid. A total of eight participants were included in this study; all received amoxicillin alone and amoxicillin with probenecid in alternating weeks, yielding 128 concentration–time profiles (16 data points per participant).

#### Modelling and simulation

PopPK analysis was performed using the non-linear mixed-effects modelling software Monolix 2024R1 (Lixoft SAS, a Simulations Plus company), employing the stochastic approximation expectation–maximization algorithm. Subsequently, Monte Carlo simulations were conducted using Simulx 2024R1 (Lixoft SAS, a Simulations Plus company) to evaluate potential probenecid-boosted amoxicillin dosing regimens suitable for OPAT. Detailed methodology for the modelling and simulation approach is presented in the Supplementary material (Supplementary information S1).

## Results

### Stability

No visual precipitation was observed in any of the amoxicillin solutions at any of the tested concentrations. Reconstitution of amoxicillin in CBS resulted in a marked elevation in pH relative to the initial diluent value (pH 7.00), with post-reconstitution pH values of 8.71, 9.03, and 9.02 for low, intermediate, and high concentrations, respectively. During refrigerated storage (2–8°C) in 0.3% citrate, pH remained relatively stable at the low concentration (ΔpH = 0.09) but declined significantly at intermediate (ΔpH = 0.34) and high concentrations (ΔpH = 0.38). Comparable pH trends were observed in WFI, whereas normal saline exhibited significant pH shifts at all concentrations (Tables S1–S3).

**Figure 1.**
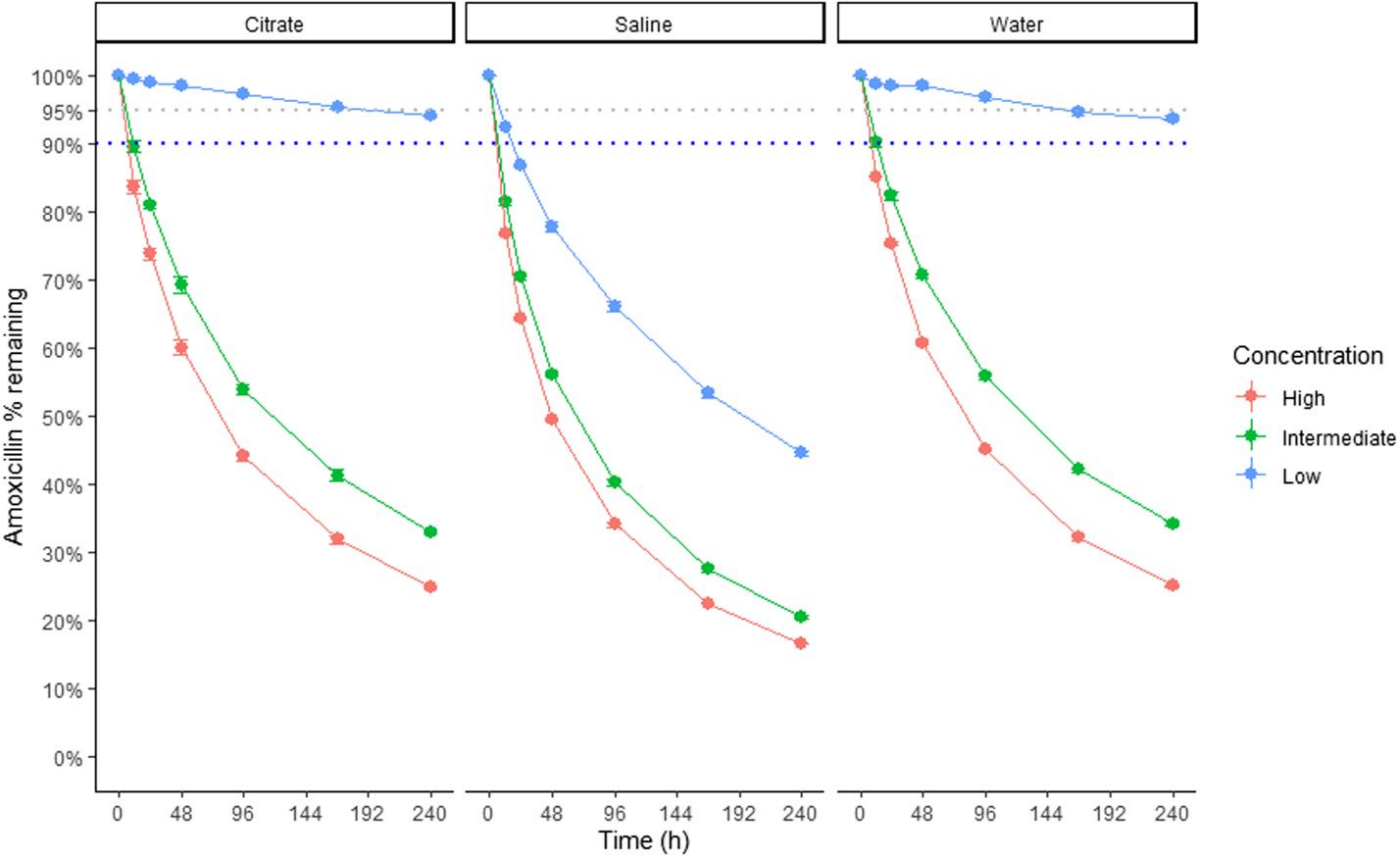
Stability of amoxicillin solution in 0.3% citrate, normal saline and water for injection during 10 days of refrigerated storage at high (66.6 mg/mL), intermediate (50 mg/mL), and low (4.17 mg/mL) concentrations.

Figure 1 illustrates the degradation of amoxicillin during refrigerated storage. In CBS and WFI, exponential degradation was observed at intermediate and high concentrations, with >10% loss within 24 h and >60% loss by day 10 (240 h). In contrast, low-concentration solutions in CBS and WFI exhibited linear degradation kinetics, retaining >95% of the initial concentration at day 7 (168 h) and >90% at day 10. In normal saline, exponential degradation occurred across all concentrations, including low concentrations, with >20% loss within 48 h.

**Figure 2.**
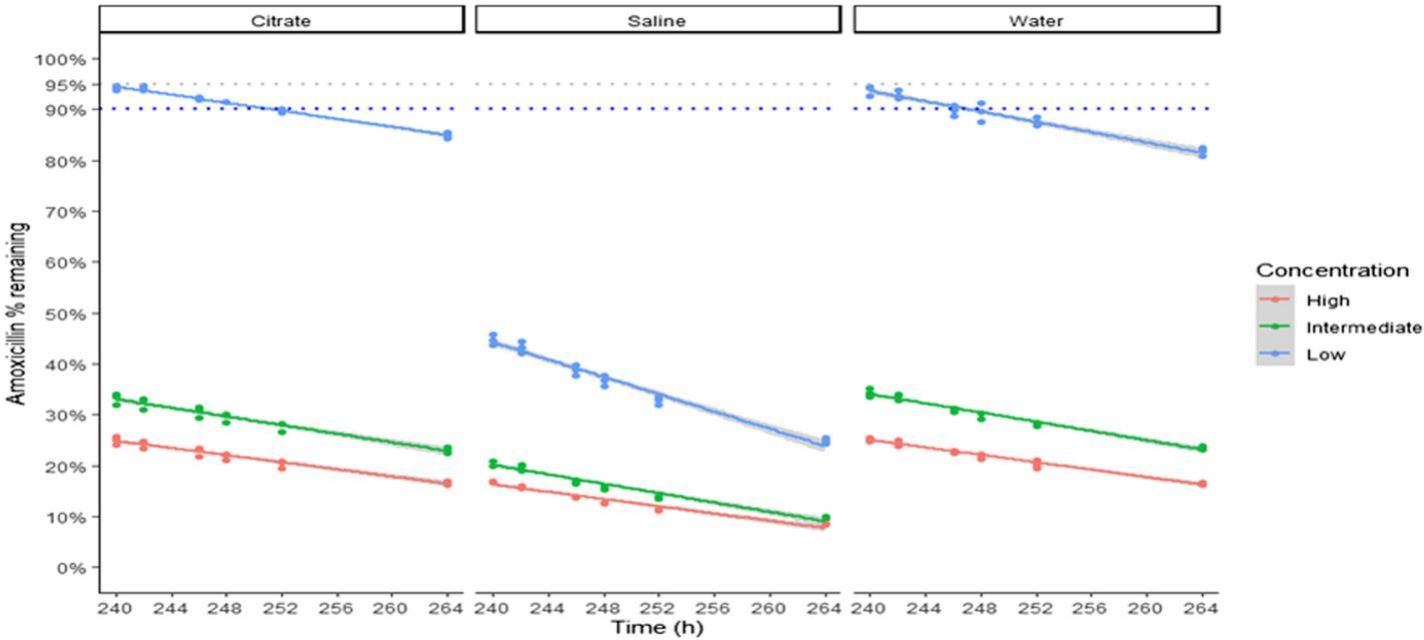
Stability of amoxicillin solution in 0.3% citrate, normal saline and water for injection during 24 h exposures to in-use temperature of 32°C following 10 days (240 h) of refrigerated storage at high (66.6 mg/mL), intermediate (50 mg/mL), and low (4.17 mg/mL) concentrations.

At the in-use temperature of 32°C, a similar degradation rate was observed across all amoxicillin concentrations in WFI and CBS (Figure 2). Amoxicillin loss exceeded 70% within 12 h at intermediate and high concentrations. At low concentration, degradation was limited to 10%–12% over the same period. In saline, degradation was rapid even at low concentrations, with >50% loss observed within 12 h.

### Model development

The PK disposition of amoxicillin was robustly characterized using a one-compartment structural model with zero-order absorption kinetics, a lag time (Tlag), and linear systemic elimination. Random effects were incorporated into Tlag, the zero-order absorption duration (Tk0), V, and CL. This model provided an adequate fit to the observed plasma concentration–time data across all treatment groups. A proportional error model best described the residual unexplained variability (RUV) in plasma amoxicillin concentrations.

Including treatment group (probenecid co-administration) as a covariate for CL markedly improved model performance, with a ΔOFV of −29.43 and a substantial decrease in interindividual variability (IIV) for CL, from 0.28 to 0.1. The final covariate relationship for CL is expressed as:


$$CL_{j}=CL_{\;pop}*\exp(\beta_{CL,GROUP}*[G])*\exp(\eta_{CL})$$

Where CL*_j_* denotes the individual CL, CL*_pop_* is the typical population value, β*_CL_*_, *GROUP*_ represents the estimated effect of treatment group; G = 1 for probenecid co-administration and G = 0 for amoxicillin alone, and *η_CL_* captures the individual-level random effect. Population-level parameter estimates derived from the final model are presented in Table 1.

**Table 1.** Estimates of the PopPK parameters of amoxicillin

Parameter	Population estimates (RSE%) [Shrinkage%]	Bootstrap median (95% confidence interval)
Fixed effects		
F_pop	0.77 (8.42)	0.76 (0.65–0.9)
Tlag_pop	0.22 (14.7)	0.22 (0.15–0.28)
Tk0_pop (h)	2.68 (7.62)	2.68 (2.42–2.96)
V_pop/F (L)	49.23 (11.7)	48.12 (39.18–59.26)
Cl_pop/F(G = 0) (L/h)	18.71 (10.1)	18.38 (15.3–22.08)
Cl_pop/F(G = 1) (L/h)	11.12 (11.8)	10.93 (8.31–14.36)
Random effects		
IIV_ Tlag	0.47 (28.6) [10.6]	0.45 (0.23–0.85)
IIV_Tko	0.26 (22.4) [7.65]	0.26 (0.18–0.34)
IIV_V	0.29 (23.8) [5.8]	0.29 (0.14–0.40)
IIV_CL	0.1 (26.6) [16.3]	0.092 (0.065–0.11)
Residual errors		
Proportional (b)	0.14 (8.53)	0.13 (0.11–0.15)

Cl_pop/F(G = 0), apparent population clearance with amoxicillin alone; Cl_pop/F(G = 1), apparent population clearance with probenecid co-administration; F, bioavailability; h, hour; IIV_CL, interindividual variability in clearance; IIV_Tlag, interindividual variability in lag time before absorption; IIV_Tk_0_, interindividual variability in zero-order absorption duration; IIV_V, interindividual variability in volume of distribution; L, litre; RSE, relative standard error; Tk_0_, zero-order absorption duration; V_pop/F, apparent population volume of distribution.

### Model evaluation

**Figure 3.**
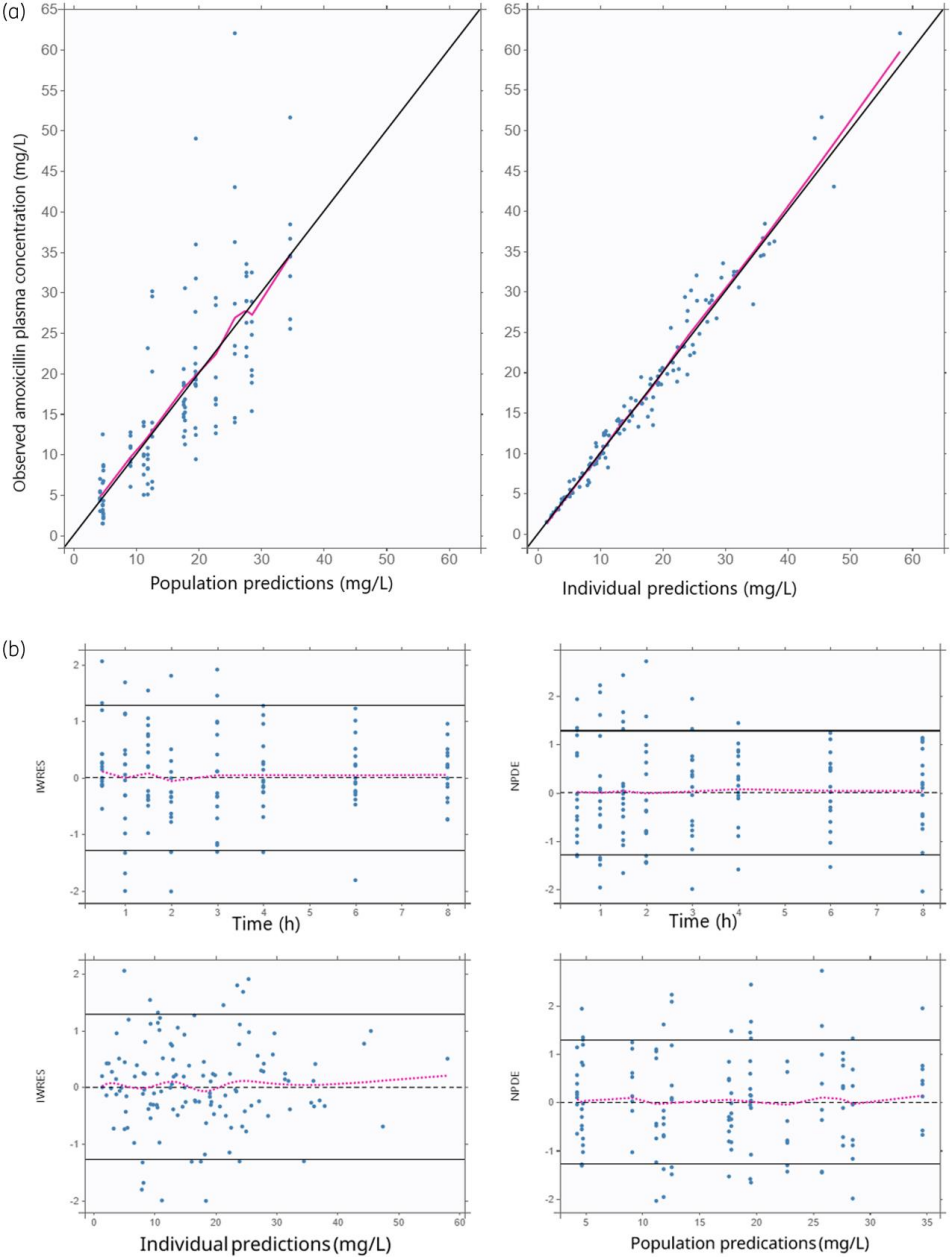
Goodness-of-fit plots for plasma concentrations of amoxicillin. (a) Observations versus population (left) and individual (right) predictions. (b) Scatter plots of the residuals.

PopPK parameter estimates were obtained with high precision, and the magnitudes of IIV and RUV were within anticipated physiological ranges (Table 1). Goodness-of-fit diagnostics, plots of observed versus both population-predicted and individual-predicted amoxicillin concentrations, and scatter plots (Figure 3) show no obvious misspecification of the structural and associated statistical models used to describe inter-individual variability and residual errors. The VPC plots indicate no model misspecification (Figure S4). Among 1000 bootstrap replicates, all runs converged successfully, and population parameter estimates from the final model closely matched the median values and 95% confidence intervals of the bootstrap results (Table 1), indicating that there are no obvious signs of model instability, overparameterization, or boundary issues.

### Monte Carlo simulation and PTA

PTA analysis using a PK/PD target of 95% fT > MIC showed that IV amoxicillin regimens administered q12h or q24h without probenecid were generally inadequate to achieve the predefined target (PTA ≥90%) across most MIC values, particularly at MICs ≥ 0.5 mg/L. PTA values for MICs ranging from 1 to 16 mg/L are presented in Table 2.

**Table 2.** PTA of IV amoxicillin regimens ± probenecid at varying MIC values, based on the PK/PD target of 95% fT > MIC

Amoxicillin dosing regimens	Infusion duration (h)	PTA for amoxicillin alone by MIC (mg/L)					PTA for amoxicillin + 1 g probenecid by MIC (mg/L)					PTA for amoxicillin + 2 g probenecid by MIC (mg/L)				
		1	2	4	8	16	1	2	4	8	16	1	2	4	8	16
1 g IV amoxicillin every 12 h	0.5	10.7	0.9	0	0	0	86.9	56.1	9.1	0	0	**99.9**	**98.4**	86	20.3	0
	2	19.9	1.6	0	0	0	**93.4**	72.1	17.6	0	0	**100**	**99.8**	**93**	32.8	0
	3	32	3.6	0	0	0	**95.3**	77.1	28.6	0.1	0	**100**	**100**	**95.5**	45.8	0
	4	36.8	6.9	0	0	0	**97**	83.6	33.3	0.3	0	**100**	**100**	**96.6**	50.7	0
1.5 g IV amoxicillin every 12 h	0.5	26.6	4.6	0.1	0.1	0	**93.6**	78	35.6	1.4	0	**100**	**99.8**	**95.5**	66.7	1.8
	2	36.9	10.2	0.1	0.1	0	**97.6**	86.1	49.8	4	0	**100**	**100**	**98.4**	78	5.7
	3	46.1	15.6	0.4	0.4	0	**98.6**	**91.2**	61.3	5.4	0	**100**	**100**	**99.4**	85.7	7.9
	4	59.9	21.2	1	1	0	**99.5**	**94.9**	70.1	10	0	**100**	**100**	**99.6**	**90.1**	11.7
2 g IV amoxicillin every 12 h	0.5	38.4	13.6	0.9	0.9	38.4	**95.9**	87.6	57.2	12	0	**100**	**99.9**	**98.3**	85.8	22.3
	2	50.8	19	1.5	1.5	50.8	**99.1**	**94**	70.1	16.5	0	**100**	**99.9**	**99.6**	**93.7**	31.7
	3	59.3	27.3	3.5	3.5	59.3	**99.2**	**96**	80.3	24.5	0	**100**	**100**	**100**	**96**	41.1
	4	71.1	36.8	5.8	5.8	71.1	**100**	**98**	86.6	34.6	0	**100**	**100**	**100**	**98**	52.7
3 g IV amoxicillin every 12 h	0.5	52.3	27.9	5.8	5.8	0	**98.8**	**94.3**	78	37.6	2.4	**100**	**99.9**	**99.9**	**96**	67
	2	65.6	38.5	10.5	10.5	0	**99.3**	**96.2**	84.7	49.6	3.6	**100**	**100**	**100**	**98**	78
	3	73.1	45.7	18	18	0	**99.5**	**97.8**	**90.5**	58.7	6.8	**100**	**100**	**100**	**98.8**	85
	4	82.6	57.8	25.4	25.4	0	**99.9**	**99.4**	**94.8**	69.1	10.1	**100**	**100**	**100**	**99.8**	89.8
4 g IV amoxicillin every 12 h	0.5	63.2	37.6	12.4	0.7	0	**99.2**	**96**	85.5	56.8	10.1	**100**	**100**	**100**	**98.8**	84.5
	2	72.4	49.5	19.9	1.5	0	**99.9**	**98.5**	**93.5**	68	17.3	**100**	**100**	**100**	**99.7**	**93.3**
	3	81	60.9	27.5	4.2	0	**100**	**99.9**	**96**	78	25.5	**100**	**100**	**100**	**99.9**	**95.3**
	4	86.7	69.5	36.3	7.7	0.1	**100**	**99.4**	**97.5**	84.3	33.5	**100**	**100**	**100**	**100**	**97.4**
6 g IV amoxicillin every 12 h	0.5	76.4	55.6	26.8	6.5	0.1	**99.7**	**98.9**	**94.7**	79.8	37.7	**100**	**100**	**100**	**99.7**	**97.2**
	2	83.3	66.7	39.6	10.5	0.1	**100**	**99.5**	**97.6**	85.8	50.6	**100**	**100**	**100**	**99.6**	**92.9**
	3	89.6	77.3	50.9	16.2	1	**100**	**99.8**	**98.9**	**92.8**	63.7	**100**	**100**	**100**	**100**	**99.5**
	4	**93**	83.5	58.5	21.7	0.8	**99.9**	**99.7**	**99.9**	**94.3**	72.2	**100**	**100**	**100**	**100**	**99.3**
2 g IV amoxicillin every 24 h	0.5	0	0	0	0	0	13.3	1.6	0	0	0	79.4	50.4	14.3	0.2	0
	2	0.1	0	0	0	0	16.2	3.3	0.1	0	0	82	56	17.5	0.4	0
	3	0.2	0	0	0	0	20	3.3	0.2	0	0	86.7	61	21.1	1	0
	4	0.5	0	0	0	0	26.5	7	0.5	0	0	86.8	67.6	28.9	1.6	0
3 g IV amoxicillin every 24 h	0.5	0.6	0.2	0	0	0	25.8	7.8	1.2	0	0	88.4	69	34.9	4.8	0.1
	2	0.5	0	0	0	0	29.7	10.7	1.3	0	0	**91.5**	75	40.6	7	0
	3	0.4	0	0	0	0	33.6	12.7	1.1	0	0	**92**	76.2	43.3	7.8	0
	4	1.1	0	0	0	0	36.1	14.5	2.4	0	0	**92.8**	77.8	47.9	10.1	0
8 g IV amoxicillin every 24 h	0.5	3.4	0.4	0	0	0	56.3	35.9	14.1	3.3	0.1	**96.3**	**91.5**	78.3	51.5	15.1
	2	4.9	0.8	0.1	0	0	58.9	39.1	17.1	3.6	0.1	**98.5**	**93.8**	81.8	55	17.7
	3	5.3	1.1	0.1	0	0	67.2	43.8	20.4	4.3	0.1	**98.4**	**94.8**	85.4	63.4	21.1
	4	6.4	1.5	0.2	0	0	70.7	46.6	23.8	5.5	0.2	**98.8**	**95.9**	88.3	64.7	25.3
12 g IV amoxicillin every 24 h	0.5	6.8	2	0.1	0	0	69.6	49.3	26.6	8	0.9	**98.6**	**95.9**	89	72.5	36.6
	2	6.4	1.9	0.2	0.1	0	70.7	53.9	30.2	8.4	0.8	**99.2**	**97.9**	**90.6**	73.6	41.4
	3	8	2.5	0.6	0	0	77.8	58.8	33.7	11.1	1.5	**99.3**	**98.3**	**92.4**	79.7	45.6
	4	11.2	4.7	1	0.1	0	76.8	60.2	37.3	14.9	2.4	**99.7**	**97.5**	**93.9**	78.6	48.8

Bold values indicate dosing regimens achieving the predefined PTA target of ≥ 90%. g, gram; IV, intravenous.

Co-administration of probenecid substantially enhanced IV amoxicillin target attainment. At a probenecid dose of 1 g, PTA ≥90% was achieved at an MIC of 1 mg/L for the 1 g q12h amoxicillin regimen (2–4 h infusion), and at an MIC of 2 mg/L for the rest of the amoxicillin regimens (q12h), including 1.5 g q12h (3–4 h infusion) and 2 g q12h (2–4 h infusion). At higher amoxicillin doses (3–6 g q12h), this effect further extended coverage to MICs of 4–8 mg/L, depending on the infusion duration (Table 2).

Increasing the probenecid dose to 2 g once daily further improved target attainment, with all q12h amoxicillin regimens achieving near-complete PTA (approximately 100%) at MICs of 1–2 mg/L. Furthermore, PTA ≥90% was achieved up to an MIC of 4 mg/L with amoxicillin 1–1.5 g q12h, up to 8 mg/L with 2–3 g q12h amoxicillin, and up to 16 mg/L with 4–6 g q12h amoxicillin, mostly irrespective of infusion duration. However, among q24h regimens, only the highest amoxicillin doses (8–12 g) achieved PTA ≥90% at MICs of 2–4 mg/L, depending on infusion duration.

## Discussion

This study demonstrates that the marked aqueous instability of amoxicillin under OPAT-relevant conditions represents a potential barrier to its administration via CI. The principal findings of this study were that: (i) none of the evaluated formulations, including both buffered and unbuffered diluents, met acceptable stability thresholds; (ii) co-administration with probenecid substantially improved IV amoxicillin exposure and PTA for 95% fT > MIC, enabling effective intermittent dosing regimens without the need for CI; and (iii) probenecid-boosted IV amoxicillin regimens represented a promising alternative OPAT dosing strategy with potential antimicrobial stewardship benefits through reduced reliance on broad-spectrum antibiotics.

The necessity for such alternative strategies is underscored by the growing interest in using amoxicillin for the management of deep-seated infections in the OPAT programmes.^23^ However, its marked instability in aqueous solutions^18,19^ does not allow prolonged (continuous) infusion of the total daily dose via portable infusion pumps, which would otherwise require multiple divided doses, thereby making outpatient practice inconvenient and limiting its clinical use. Poor aqueous stability is consistently reported in the literature,^20^ and the present study reinforces this, extending previous observations to buffered and unbuffered diluents.

Refrigerated storage did not adequately maintain amoxicillin concentration across all doses and solvents tested, whether buffered or unbuffered. Degradation was concentration-dependent in both the CBS and WFI. High and intermediate concentrations declined exponentially, losing more than half of their initial concentration, whereas low concentrations degraded more slowly and linearly. In saline, degradation occurred rapidly across all concentrations. Losses exceeded 20% within 48 h of refrigerated storage at the low concentration, with even greater degradation observed at the intermediate and high concentrations. Of note, these results differ from those reported by Arensdorff *et al*.,^23^ who reported less than 10% degradation over the same period when 6 g of amoxicillin was reconstituted in 240 mL of saline, a discrepancy that may be attributable to methodological differences, such as analytical techniques.

At clinically relevant in-use temperatures (32°C), degradation accelerated markedly and remained concentration-dependent in both CBS and WFI. Consistent with previous reports,^10,19,24^ high-concentration solutions lost more than 70% of their initial concentration within 12 h, whereas low-concentration preparations exhibited greater stability, with only 10%–12% loss. This phenomenon is likely driven by increased molecular interactions at higher concentrations, promoting autocatalytic self-ammonolysis and the formation of inactive dimers and polymers.^25^ In saline, degradation was rapid even at low concentrations, with nearly 50% loss observed within 12 h, further underscoring the unsuitability of this diluent for continuous amoxicillin administration. However, we note that these findings differ from some studies, including those by d’Huart *et al*.,^26^ who reported ≥90% stability at 12.5 mg/mL over 12 h, and Arlicot *et al*.,^10^ who described relative stability in saline at concentrations ≤40 mg/mL at 20°C and 35°C. Nevertheless, several critical methodological limitations, such as poor design lacking sequential refrigerated storage and in-use exposure data (non-compliance with YCD requirements) and the use of assays not validated as stability-indicating methods (i.e. not validated to distinguish amoxicillin from its degradation products),^19^ may have contributed to the discrepancy.

Notably, none of the formulations tested in this study met the YCD acceptance range (95%–105% of initial concentration). Given this challenge, we investigated the co-administration of probenecid as a viable alternative strategy to optimize amoxicillin exposure without the need for CI. Using a PK/PD target of 95% fT > MIC, our study demonstrated that co-administration of probenecid substantially increases amoxicillin exposure and yields a favourable PTA across the susceptible MIC range for common pathogens of interest. For instance, IV amoxicillin 3 g q12h (3–4 h infusion) combined with oral probenecid 1 g once daily achieves PTA exceeding 90% for pathogens with MICs up to 4 mg/L. Increasing the amoxicillin dose to 4 g q12h (2–4 h infusion) or 6 g q12h standard infusion while maintaining probenecid at 1 g once daily did not yield further improvement in target attainment, suggesting that the 3 g q12h regimen plus probenecid 1 g once daily regimen may be sufficient against amoxicillin-susceptible pathogens with clinical breakpoints of ≤4 mg/L (e.g. *Enterococcus* spp.).

For pathogens with higher MICs, amoxicillin 6 g q12h as a 3-h extended infusion, with probenecid 1 g once daily may be required to maintain adequate PTA against pathogens with clinical breakpoints up to 8 mg/L (e.g. *Enterobacterales*). Furthermore, simulating probenecid at 2 g once daily, reflecting clinical scenarios in which this dose is used as a β-lactam enhancer,^21^ demonstrated that IV amoxicillin (1 g q12h as 2 h infusion or 1 .5 g q12h standard infusions) achieved adequate PTA at MICs of 4 mg/L; while increasing amoxicillin doses and/or infusion regimens enable to achieve target attainment (>90%) against pathogens with MIC values up to 16 mg/L (Table 2). These highlight the potential of probenecid-boosted amoxicillin regimens as viable dosing strategies for the targeted therapy of infections caused by susceptible pathogens with relatively high MICs.

The PK enhancement observed with probenecid co-administration results from inhibition of organic anion transporters (OAT1 and OAT3), which blocks renal tubular secretion of amoxicillin and prolongs systemic exposure.^21^ This mechanism significantly increases serum concentrations relative to amoxicillin monotherapy^27^ and enhances %fT > MIC, the primary PK/PD index associated with β-lactam antibiotic efficacy.^28^ The PK interaction of probenecid with β-lactam antibiotics is well documented and has been reviewed in detail elsewhere^28^; clearly, it substantially increases exposure with observed higher AUC, %fT > MIC and PTA across multiple β-lactam antibiotics.

Probenecid-enhanced amoxicillin therapy aligns closely with antimicrobial stewardship principles by enabling narrow-spectrum, pathogen-directed treatment. Amoxicillin’s targeted activity minimizes disruption to the host microbiota, thereby reducing the risk of *Clostridioides difficile* infection and antimicrobial resistance development.^29,30^ This approach supports antimicrobial stewardship by reducing reliance on broad-spectrum agents, which are often selected for dosing convenience rather than clinical necessity.^31,32^ As OPAT programmes continue to evolve, the use of IV amoxicillin may represent a clinically sound approach that optimizes the management of serious infections in community settings while preserving the use of broad-spectrum antimicrobials and limiting ecological harm.

Within the broader context of narrow-spectrum OPAT strategies, this study demonstrates that an amoxicillin-probenecid boosted regimen could provide additional treatment options for streptococcal and enterococcal infections, alongside other agents such as benzylpenicillin and ampicillin. Citrate-buffered benzylpenicillin demonstrates stability (>95%) adequate for CI and is suitable for many such infections.^33,34^ Ampicillin, on the other, despite being a useful agent for enterococcal infections, is limited by its shorter in-use stability window at 32°C,particularly at higher concentrations.^35^ Of note, an oral amoxicillin–probenecid combination may also represent a promising alternative when oral treatment is acceptable. The POET sub-study reported a 75%–85% PTA against enterococci with high-dose oral amoxicillin alone^4^; suggesting that the addition of probenecid could further enhance target attainment; this warrants prospective evaluation in clinical studies.

This study has some potential limitations. First, although the study aimed to reflect clinically used buffer systems, only a 0.3% citrate buffer was evaluated, which did not provide adequate buffer capacity to ensure pH control. Future studies should evaluate clinically acceptable buffer strengths that offer adequate buffer capacity. Second, the *in silico* PK analysis was based on data from healthy volunteers; whilst these may reasonably represent OPAT patients who are medically stable, ideally data from patients should be used to maximize the predictive performance of the PK model. Third, the model utilized data from a limited number of subjects and may not fully capture inter-individual PK variability. Fourth, in the absence of both oral and IV data for amoxicillin co-administered with probenecid, our model derived a theoretical numeric estimate for F, although this was subsequently validated by a literature value. Future prospective studies should estimate this in patients receiving both oral and IV amoxicillin co-administered with probenecid. Fifth, although oral probenecid may be convenient for outpatient use, dose-dependent GI adverse effects (e.g. nausea, vomiting, and GI upset; reported in only 78 of 2502 patients receiving probenecid 3 g/day), which can be lessened with food, may affect tolerability and compliance.^36^ In addition, potential interactions with drugs that undergo tubular secretion may complicate the use of probenecid in some patients.

In conclusion, despite the clinical demand for amoxicillin in OPAT, particularly for enterococcal and streptococcal infections, its instability under OPAT-relevant conditions remains a significant barrier to CI. Co-administration with probenecid presents a pharmacologically viable alternative strategy that extends systemic exposure and maintains therapeutic concentrations without requiring CI. Future prospective randomized controlled trials should evaluate long-term clinical outcomes, safety, tolerability, PD target attainment, microbiological response, and infection-specific efficacy to establish the broader clinical applicability of this approach.

## Supplementary Material

dlag165_Supplementary_Data

## Transparency declarations

F.B.S. received a research grant from Pfizer 418 (2022–23), Gilead Australia Fellowship (2023) and British Society for Antimicrobial Chemotherapy, BSAC (2021–23). J.A.R. is a consultant/advisory board of Qpex (2022), Gilead (2022), Advanz Pharma (2022), Pfizer (2020), Sandoz (2020), Wolters Kluwer (2021), MSD (2021), and Summit Pharma (2021). J.A.R. received funding from Pfizer (2023), MSD (2022), Gilead (2022), and Cipla (2021) to speak at conferences. J.A.R. received industry grants from Pfizer (2023), QPEX (2021), and BSAC (2020–23). M.G. is a consultant/advisory board of Pfizer (2023) and Menarini (2023). M.G. is a co-lead of the BSAC OPAT Initiative. R.A.S. is a consultant/advisory board and/or speaker for Pfizer, Menarini, Shionogi, and Napp (2023–24). R.A.S. is a co-lead of the BSAC OPAT initiative.

## Supplementary data

Figures S1–S4 and Tables S1–S3 are available as Supplementary data at *JAC-AMR* Online.
