# Supplementary material for: Optimizing intravenous amoxicillin for outpatient parenteral antimicrobial therapy
Source: JAC Antimicrob Resist. 2026 Aug 6;8(4):dlag165. doi: 10.1093/jacamr/dlag165 (PMC13445613; doi:10.1093/jacamr/dlag165)
Supplement: Supplementary file 1 [file dlag165_supplementary_data.docx]

**Supplementary materials**

Optimising intravenous amoxicillin for outpatient parenteral antimicrobial therapy

**Supplementary information 1: Methods: - Pharmacokinetic modelling and Monte Carlo dosing simulations**

**Model development**

PopPK analysis was performed using the non-linear mixed-effects modelling software Monolix 2024R1 (Lixoft SAS, a Simulations Plus company), employing the stochastic approximation expectation–maximisation (SAEM) algorithm. Candidate structural models included one- and two-compartment models with zero- or first-order absorption and first-order elimination. Models from Monolix inbuilt library with IV or extravascular input were used to derive a numerical estimate for F as free parameter with literature informed initial parameter estimates and subsequent validation of acceptability in reference to literature value.

Individual PK parameter estimates were assumed to follow a log-normal distribution, except for bioavailability (F), which was modelled assuming a logit-normal distribution, restricting F values between 0 and 1. Interindividual variability (IIV) in key PK parameters, namely apparent clearance (CL/F), apparent volume of distribution (V/F), estimated population lag time before absorption (Tlag), and zero-order absorption duration (Tk₀), was described using an exponential random-effects model:

***θj* = *θp* × exp (*ηj)***

Where *θj* represents the parameter estimate for the *j*th individual, *θ*p is the typical population value, and *ηj* is the individual deviation that follows a normal distribution with mean zero and variance *ω*^2^. Residual unexplained variability (RUV) was explored using constant, additive, proportional, or combined additive–proportional error models. Model selection was guided by the accuracy and precision of parameter estimates, diagnostic goodness-of-fit (GOF) plots, visual predictive checks (VPCs), changes in the objective function value (OFV), and the Bayesian Information Criterion (BIC).

**Covariate analysis**

Following stabilisation of the base structural model, the treatment group (with versus without probenecid co-administration) was evaluated as a categorical covariate on key PK parameters, including CL and volume of distribution (V). Covariate effect (limited to treatment group) was retained in the final model if a statistically significant reduction in the objective function value (−2LL decrease > 3.84; P < 0.05) was observed.

**Model evaluation**

Model evaluation was performed using a combination of graphical and numerical diagnostics to ensure robustness and predictive reliability. GOF assessments, including plots of observed versus population-predicted and individual-predicted concentrations, were examined to evaluate model adequacy. Numerical criteria included the OFV, precision of parameter estimates, IIV, and RUV. Additionally, the biological plausibility of parameter estimates, and covariate relationships was carefully assessed. Model stability and predictive performance were further validated through nonparametric bootstrap resampling (1,000 runs). For each parameter, the median, 2.5th, and 97.5th percentile values derived from the bootstrap distributions were compared with the corresponding estimates from the original dataset. In addition, the numerical estimate for F was only deemed acceptable when comparable to literature reported values given it cannot be uniquely identified from oral data alone.

**Dosing simulations and PTA**

Monte Carlo simulations were conducted using Simulx 2024R1 (Lixoft SAS, a Simulations Plus company) to evaluate potential probenecid-boosted amoxicillin dosing regimens suitable for OPAT. These simulations were based on the final PK model developed from orally administered concentration–time data. In a comprehensive simulation involving 1,000 virtual patients, a range of IV amoxicillin regimens, 1, 1.5, 2, 3,4 and 6 g every 12 hours (q12h) and 2,3,8 and 12 g every 24 hours (q24h), were evaluated across infusion durations of 0.5, 2, 3, and 4 hours, with and without co-administration of 1 g oral probenecid once daily. This probenecid dosing regimen was selected, partly based on the availability of amoxicillin-probenecid interaction data ^1^ and the evidence that 1 g once daily is the typical dosing regimen used as a beta-lactam enhancer. ^2, 3^ The decision to also evaluate 2 g of probenecid was based on PK data showing that increasing the dose from 1 g to 2 g raised the AUC of certain beta-lactam antibiotics, corresponding to an approximate 36% reduction in CL,^4^ and acceptable tolerability at doses up to 2 g/day.^3^ The PTA was calculated across a range of MIC thresholds (0.03125 to 32 mg/L), using a PK/PD target of 95% *f*T_>MIC_ ^5-8^over 24 hours. Regimens achieving ≥90% PTA at clinically relevant MIC breakpoints were deemed optimal therapeutic strategies.

**Supplementary information 2: Results: - Supplementary Tables**

| **Table S1.** Observed change in pH over time for amoxicillin reconstituted with 0.3 % sodium citrate buffer pH=7 at low (1 g/240 mL), intermediate (12 g/240 mL), and high concentrations (16 g/240 mL) | | | | | | | |
| --- | --- | --- | --- | --- | --- | --- | --- |
| Temperature Condition | Time | Observed pH and change in pH by concentrations | | | | | |
|  |  | Low Concentrations | | Intermediate Concentration | | High Concentrations | |
|  |  | Observed pH | Δ pH | Observed pH | Δ pH | Observed pH | Δ pH |
| Fridge Storage (5 ± 3 °C) | 0 | 8.71 ± 0.00 | 0.00 | 9.03 ± 0.00 | 0.00 | 9.02 ± 0.00 | 0.00 |
|  | 24 | 8.59 ± 0.01 | 0.12 | 8.82 ± 0.02 | 0.21 | 8.78 ± 0.01 | 0.24 |
|  | 48 | 8.62 ± 0.03 | 0.09 | 8.81 ± 0.01 | 0.22 | 8.79 ± 0.01 | 0.23 |
|  | 96 | 8.55 ± 0.00 | 0.16 | 8.71 ± 0.01 | 0.32 | 8.67 ± 0.01 | 0.35 |
|  | 168 | 8.58 ± 0.01 | 0.13 | 8.69 ± 0.01 | 0.34 | 8.64 ± 0.01 | 0.38 |
|  | 240 | 8.62 ± 0.01 | 0.09 | 8.69 ± 0.01 | 0.34 | 8.64 ± 0.01 | 0.38 |
| In-use temperature (32 °C) | 242 | 8.61 ± 0.01 | 0.10 | 8.65 ± 0.01 | 0.38 | 8.59 ± 0.01 | 0.43 |
|  | 246 | 8.47 ± 0.01 | 0.24 | 8.51 ± 0.02 | 0.52 | 8.45 ± 0.01 | 0.57 |
|  | 248 | 8.47 ± 0.01 | 0.24 | 8.49 ± 0.01 | 0.54 | 8.44 ± 0.01 | 0.58 |
|  | 252 | 8.43 ± 0.01 | 0.28 | 8.43 ± 0.01 | 0.60 | 8.38 ± 0.01 | 0.64 |
|  | 264 | 8.35 ± 0.01 | 0.36 | 8.32 ± 0.01 | 0.71 | 8.27 ± 0.01 | 0.75 |

| **Table S2.** Observed change in pH over time for amoxicillin reconstituted with normal saline at low (1 g/240 mL), intermediate (12 g/240 mL), and high concentrations (16 g/240 mL) | | | | | | | |
| --- | --- | --- | --- | --- | --- | --- | --- |
| Temperature Condition | Time | Observed pH and change in pH by concentrations | | | | | |
|  |  | Low Concentrations | | Intermediate Concentration | | High Concentrations | |
|  |  | Observed pH | Δ pH | Observed pH | Δ pH | Observed pH | Δ pH |
| Fridge Storage (5 ± 3 °C) | 0 | 8.74 ± 0.00 | 0.00 | 8.89 ± 0.00 | 0.00 | 8.93 ± 0.00 | 0.00 |
|  | 24 | 8.61 ± 0.01 | 0.13 | 8.70 ± 0.00 | 0.19 | 8.69 ± 0.01 | 0.24 |
|  | 48 | 8.60 ± 0.01 | 0.14 | 8.69 ± 0.01 | 0.20 | 8.67 ± 0.01 | 0.26 |
|  | 96 | 8.45 ± 0.02 | 0.29 | 8.53 ± 0.01 | 0.36 | 8.52 ± 0.00 | 0.41 |
|  | 168 | 8.38 ± 0.03 | 0.36 | 8.48 ± 0.02 | 0.41 | 8.47 ± 0.01 | 0.46 |
|  | 240 | 8.32 ± 0.03 | 0.42 | 8.43 ± 0.02 | 0.46 | 8.42 ± 0.01 | 0.51 |
| In-use temperature (32 °C) | 242 | 8.25 ± 0.01 | 0.49 | 8.35 ± 0.02 | 0.54 | 8.33 ± 0.00 | 0.60 |
|  | 246 | 8.09 ± 0.01 | 0.65 | 8.21 ± 0.01 | 0.68 | 8.17 ± 0.01 | 0.76 |
|  | 248 | 8.06 ± 0.01 | 0.68 | 8.17 ± 0.01 | 0.72 | 8.13 ± 0.00 | 0.80 |
|  | 252 | 7.92 ± 0.12 | 0.82 | 8.09 ± 0.01 | 0.80 | 8.05 ± 0.00 | 0.88 |
|  | 264 | 7.76 ± 0.00 | 0.98 | 7.90 ± 0.02 | 0.99 | 7.85 ± 0.02 | 1.08 |
| **Table S3**. Observed change in pH over time for amoxicillin reconstituted with water for injection at low (1 g/240 mL), intermediate (12 g/240 mL), and high concentrations (16 g/240 mL) | | | | | | | |
| Temperature Condition | Time | Observed pH and change in pH by concentrations | | | | | |
|  |  | Low Concentrations | | Intermediate Concentration | | High Concentrations | |
|  |  | Observed pH | Δ pH | Observed pH | Δ pH | Observed pH | Δ pH |
| Fridge Storage (5 ± 3 °C) | 0 | 8.97 ± 0.00 | 0.00 | 8.97 ± 0.00 | 0.00 | 8.99 ± 0.00 | 0.00 |
|  | 24 | 8.86 ± 0.00 | 0.11 | 8.84 ± 0.00 | 0.13 | 8.82 ± 0.01 | 0.17 |
|  | 48 | 8.91 ± 0.02 | 0.06 | 8.87 ± 0.01 | 0.10 | 8.83 ± 0.01 | 0.16 |
|  | 96 | 8.85 ± 0.01 | 0.12 | 8.77 ± 0.01 | 0.20 | 8.72 ± 0.01 | 0.27 |
|  | 168 | 8.89 ± 0.01 | 0.08 | 8.75 ± 0.00 | 0.22 | 8.67 ± 0.01 | 0.32 |
|  | 240 | 8.96 ± 0.04 | 0.01 | 8.73 ± 0.05 | 0.24 | 8.67 ± 0.01 | 0.32 |
| In-use temperature (32 °C) | 242 | 8.91 ± 0.02 | 0.06 | 8.7 ± 0.01 | 0.27 | 8.62 ± 0.01 | 0.37 |
|  | 246 | 8.73 ± 0.01 | 0.24 | 8.56 ± 0.01 | 0.41 | 8.48 ± 0.01 | 0.51 |
|  | 248 | 8.71 ± 0.01 | 0.26 | 8.53 ± 0.01 | 0.44 | 8.44 ± 0.01 | 0.55 |
|  | 252 | 8.67 ± 0.02 | 0.30 | 8.48 ± 0.01 | 0.49 | 8.40 ± 0.01 | 0.59 |
|  | 264 | 8.52 ± 0.00 | 0.45 | 8.33 ± 0.01 | 0.64 | 8.27 ± 0.01 | 0.72 |

**Supplementary information 3: Results: - Supplementary Figures**

**Degradation in citrate**

Figure S1. Comparison of degradation in sodium citrate at medium amoxicillin concentration. At t=0 h (black), amoxicillin (retention time = 1.9 min) is 87.7% of integrated area. At t=264 h (pink), amoxicillin is 21.9% of integrated area

**Degradation in saline**

Figure S2. Comparison of degradation in saline at a medium amoxicillin concentration. At t=0 h (black), amoxicillin (retention time = 1.9 min) is 82.3% of integrated area. At t=264 h (pink), amoxicillin is 7.7% of integrated area.

**Degradation in water**

Figure S3. Comparison of degradation in water at a medium amoxicillin concentration. At t=0 h (black), amoxicillin (retention time = 1.9 min) is 88.5% of integrated area. At t=264 h (pink), amoxicillin is 22.3% of integrated area

Figure S4: Visual predictive check (VPC) plot for amoxicillin plasma concentrations

**References for supplementary information 1**

1.Barbhaiya R, Thin R, Turner P et al. Clinical pharmacological studies of amoxycillin: effect of probenecid. *British Journal of Venereal Diseases,* 1979; **55**: 211-3. <https://doi.org/10.1136/sti.55.3.211>

2.Wilson RC, Arkell P, Riezk A et al. Addition of probenecid to oral β-lactam antibiotics: A systematic review and meta-analysis. *Journal of Antimicrobial Chemotherapy* 2022; **77**: 2364-72. <https://doi.org/10.1093/jac/dkac200>

3.Robbins N, Koch SE, Tranter M et al. The history and future of probenecid. *Cardiovascular toxicology* 2012; **12**: 1-9. <https://doi.org/10.1007/s12012-011-9145-8>

4.Vlasses P, Holbrook A, Schrogie J et al. Effect of orally administered probenecid on the pharmacokinetics of cefoxitin. *Antimicrobial Agents and Chemotherapy* 1980; **17**: 847-55. <https://doi.org/10.1128/aac.17.5.847>

5.Onufrak NJ, Forrest A, Gonzalez D. Pharmacokinetic and pharmacodynamic principles of anti-infective dosing. *Clinical therapeutics* 2016; **38**: 1930-47. <https://doi.org/10.1016/j.clinthera.2016.06.015>

6.Sanz-Codina M, van Os W, Pham AD et al. Target-site cefiderocol pharmacokinetics in soft tissues of healthy volunteers. *Journal of Antimicrobial Chemotherapy* 2024; **79**: 3281-8. <https://doi.org/10.1093/jac/dkae359>

7.van den Broek AK, van Schip A, Visser CE et al. Population pharmacokinetic/pharmacodynamic target attainment of ceftriaxone 2 g once daily in non‐critically ill hospitalized adult patients during the acute phase of infection. *British Journal of Clinical Pharmacology* 2023; **89**: 3262-72. <https://doi.org/10.1111/bcp.15819>

8.Lau WK, Mercer D, Itani KM et al. Randomized, open-label, comparative study of piperacillin-tazobactam administered by continuous infusion versus intermittent infusion for treatment of hospitalized patients with complicated intra-abdominal infection. *Antimicrobial agents and chemotherapy* 2006; **50**: 3556-61. <https://doi.org/10.1128/aac.00329-06>
